# Supplementary material for: Modelling the species-area relationship using extreme value theory
Source: Nat Commun. 2025 Apr 30;16:4045. doi: 10.1038/s41467-025-59239-7 (PMC12041200; doi:10.1038/s41467-025-59239-7)
Supplement: Supplementary file 1 — Supplementary Information [file 41467_2025_59239_MOESM1_ESM.docx]

Supplementary Information for

**Modelling the species-area relationship using extreme value theory**

Luís Borda-de-Água, M. Manuela Neves, Luise Quoss, Stephen P. Hubbell, Filipe S. Dias, Henrique M. Pereira

**Supplementary Note 1: The exact and asymptotic distributions of the minima**

As discussed in the main text, the species-area relationship (SAR) is determined by the distances from the focal point of sampling to the nearest individual of each species. Consequently, when conducting a complete census of a community, the corresponding SAR can be constructed by incrementally adding one to the species count each time an individual of a species not previously encountered is recorded. However, in most cases, the precise location of the closest individual to the focal point is uncertain, as we only have a sample rather than a complete census of the community. Nonetheless, we can infer its position by estimating the distribution of the minima of the corresponding distance distribution. To accomplish this, we use order statistics^1^ and extreme value theory^2–4^ for minima. A brief overview of these approaches follows.

If *M*_1_ represents the minimum of a sequence of *n* independent and identically distributed (i.i.d.), or possibly stationary, weakly dependent, random variables {*R*_1_, *R*_2_,…, *R_n_*} with a common unknown cumulative distribution function (cdf) *F*(*r*) (referred to as the parent distribution), then the cdf of the distribution of the minima *M*_1_, *L_n_*(*r*), is given by

$$L_{n}\left( r \right)=1-\left( 1-F\left( r \right) \right)^{n}. (S1.1)$$

This expression provides the exact (i.e. non-asymptotic) characterization of the distribution of the minima^1^. As *F*(*r*) is unknown, it is advantageous to derive an approximate distribution, denoted as *L*, which is obtained in the limit when *n*→∞. However, when *n*→∞ the distribution becomes highly unstable and *L_n_*(*r*) becomes degenerate, that is, the limiting density becomes concentrated into a single point^2–4^. To address this issue, some authors in the first part of the 20^th^ century^5–7^ showed that after a suitable normalization, it is possible to obtain a non-degenerated limit distribution. A frequently used (linear) transformation^3,4^ is $M_{1}^{*}=(M_{1}-b_{n})/a_{n}$), where $\{a_{n}\}\in\mathbb{R}^{+}$ and $\{b_{n}\mathbb{\}\in R}$. The sequences *a_n_* and *b_n_* should be chosen in such way that as *n*→∞, the expression S1.1 is no longer a degenerate distribution, that is, the following limit exists

$$L\left( z \right)=\lim_{n\to\infty}L_{n}\left( a_{n}z+b_{n} \right)=\lim_{n\to\infty} \left( 1-\left[ \left( 1-F\left( a_{n}z+b_{n} \right) \right) \right]^{n} \right). (S1.2)$$

An important result is that whenever the above condition is verified, the asymptotic distribution *L*(*r*) belongs to one of the three families: reverse Gumbel, reverse Fréchet, or Weibull^3,4^. Moreover, these three distribution families can be combined into a unified expression, known as the generalized extreme value distribution, denoted as *GEV_m_*, and expressed as

$${GEV}_{m}\left( z \right)=$$

$$=\left\{ \begin{matrix} 1-\exp\left\{ -\left[ 1-\xi\left( \frac{z-\mu}{\sigma} \right) \right]^{-\frac{1}{\xi}} \right\}, 1-\xi\left( \frac{z-\mu}{\sigma} \right)>0, if \xi\neq0 \\ 1-\exp\left\{ -\exp\left\{ \left( \frac{z-\mu}{\sigma} \right) \right\} \right\}, z\mathbb{\in R,}if \xi=0, \end{matrix} \right.(S1.3)$$

where $\mu\mathbb{\in R}$, *σ*>0 and $\xi\mathbb{\in R}$ are the location, scale and shape parameters, respectively. The lower branch can be derived as the limit of the upper branch when *ξ* →0. For *ξ* <0, the above equation simplifies to the Weibull distribution, for *ξ* =0 to the reverse Gumbel, and for *ξ* >0 to the reverse Fréchet distribution^3,4^. Given the significance of the Weibull distribution, *W*(*r*), in this work, we present it explicitly here

$$W\left( r \right)=1-\exp\left( -\left( \frac{r-\lambda}{\delta} \right)^{\beta} \right), r\geq\lambda(S1.4)$$

which is equivalent to the upper branch of the *GEV_m_* (S1.3) with the following substitutions: *β*=-1/*ξ*, *δ*=-*σ/ξ* and *λ*=*μ+σ/ξ*.

The previous results can also be stated as follows. For a distribution with cdf *F*(*r*), after applying the transformation given by expression S1.2, and assuming the limit exists, the distribution of minima will fall into one of three types: reverse Gumbel, reverse Fréchet or a Weibull. These three distributions constitute the *domain of attraction* for the minima. That is, under the linear transformation, S1.2, the distribution of the minima will converge to one of these three limiting distributions. Moreover, the distributions can be combined into a single distribution call the generalized extreme value (GEV) distribution, which is particularly useful when fitting real data^3^.

The EVT results are somewhat analogous to those of the central limit theorem (CLT) which states that, under certain conditions, the distribution of the sample mean approaches a normal distribution. However, while the CLT deals with a measure of the central tendency of the distribution, EVT focuses on the extreme values of the distribution, of which we are interested here in the minima. Table S1 outlines the distributions used in this work with their respective minimal domains of attraction. For further details on the domains of attraction for maxima and minima of other common distributions, see Castillo et al.^4^, page 207, their table 9.5.

**Table S1 | Minimal domains of attraction of the distributions discussed in this manuscript**

| Parent distribution | Minimal Domain of attraction |
| --- | --- |
| Cauchy | Reverse Fréchet |
| Normal | Reverse Gumbel |
| Rayleigh | Weibull |
| Rice | Weibull |

*The asymptotic distribution of the minima of the Rayleigh distribution*

The Rice distribution is the distribution of distances of an isotropic bivariate normal distribution, and it was used in the simulations presented in the main text. The Rayleigh distribution is a specific case of the Rice distribution that arises when an isotropic bivariate normal distribution is centred at the origin^8^. Its corresponding distribution of minima can be derived analytically, thus providing an approximation for the SAR when all species have ranges centred at the origin, which according to the discussion in the main text, contribute to Phase I.

The cdf of the Rayleigh distribution is

$$F\left( r \right)=1-\exp\left( -\frac{r^{2}}{{2\sigma}^{2}} \right), r\geq0 (S1.5)$$

where *σ* >0 is the scale parameter. To derive the asymptotic distribution of its minima, one possible linear transformation of variables is *r*=*a_n_z* + *b_n_* with $a_{n}=\sqrt{\frac{2}{n}}$ and *b_n_*=0. Note that this choice of *a_n_* and *b_n_* is not unique. However, a crucial fact is that whenever a non-degenerated distribution for the minima is obtained, the shape parameter is the same regardless of the choice of *a_n_* and *b_n_*, though the location and scale parameters may vary^3^. Substituting *F*(*r*) into expression S1.2, we obtain

$$L\left( z \right)=\lim_{n\to\infty} \left( 1-\left[ \exp\left( -\frac{\left( a_{n}z+b_{n} \right)^{2}}{2\sigma^{2}} \right) \right]^{n} \right), (S1.6)$$

and using the aforementioned *a_n_* and *b_n_*, the asymptotic distribution of the minima becomes

$$L\left( z \right)=1-\exp\left[ -\left( \frac{z}{\sigma} \right)^{2} \right], (S1.7)$$

yielding a Weibull distribution with location parameter *λ*=0, scale parameter *σ* and shape parameter 2. Note that this distribution pertains to the distribution of the minima of the transformed variable *Z*=(*R* - *b_n_*)/*a_n_*. Because we have specified that $a_{n}=\sqrt{\frac{2}{n}}$ and *b_n_*=0, the cdf of the minima of the distances can be explicitly derived as

$$L\left( r;\sigma,n \right)=1-\exp\left( -\left( \frac{r}{a_{n}\sigma} \right)^{2} \right)=1-\exp\left( -\left( \frac{r}{\sigma\sqrt{2/n}} \right)^{2} \right). (S1.8)$$

This corresponds to a Weibull distribution with scale parameter $\sigma\sqrt{2/n}$ and shape parameter *β*= 2, or equivalently, to the *GEV_m_* parameter *ξ*=-1/*β=*-0.5. The significance of this finding lies in its relationship with the results of the simulations described in the main text. The simulations showed that the species contributing to Phase I exhibit distributions of minima with *ξ*=-0.5, as illustrated in Fig. 3 of the main text. This suggests that the species contributing to Phase I have ranges that are roughly centred around the focal point, and that the distributions of distances can be approximated by Rayleigh distributions.

Additionally, the assumption that distributions of distances following a Rayleigh distribution leads to the observed slope of 1 for Phase I in a double logarithmic plot (Fig. 2 of the main text). To obtain this result, substitute (S1.8) into

$$S\left( A \right)=\sum_{i=1}^{S_{T}} L\left( A;\mu_{i},\sigma_{i},\xi_{i} \right), (S1.9)$$

or

$$S\left( A \right)=\sum_{i=1}^{S_{T}} \left[ 1-\exp\left( -\left( \frac{r}{\sqrt{2/{n_{i}}}\sigma_{i}} \right)^{2} \right) \right]. (S1.10)$$

If *r* << $\sqrt{2/{n_{i}}} \sigma_{i}$, expanding the exponential term in Taylor series and retaining only the first term results in

$$S=cA, (S1.11)$$

where $c=\frac{1}{\pi}\sum_{i=1}^{S_{T}} \frac{n_{i}}{2\sigma_{i}^{2}}$, implying a slope of 1 in a double logarithmic plot. This result is independent of the distribution of the scale parameter, *σ*, or of the number of individuals, *n*.

**Supplementary Note 2: The Disk Model**

In this section we derive the species-area relationship (SAR) by making the extreme assumption that species locations possess spatial distributions represented by uniform continuous disks (see Fig. S1). We refer to this model as the “disk model.” This model is applicable when the stochastic knowledge of the minima is not relevant, which happens when the plausible range of values of the distribution of the minimum distance to the centre of the distribution is very small compared to the sampled areas. We posit the existence of *S_T_* species with spatial distributions characterized by continuous disks with a radius of 2*σ_d_*, and centroids that are uniformly randomly distributed in a region with total area $A_{T}=R_{T}^{2}$. While this model shares similarities with the one used by Allen and White^9^, our emphasis in this derivation is on elucidating the relationship between the location of a species’ range and its minimum distance to the focal point.

Starting from the focal point, a species is detected once the border of its circle is reached, and the count of species increases by one with each detection. This process can be represented mathematically as the sum of Heaviside (step) functions. For a given species, a


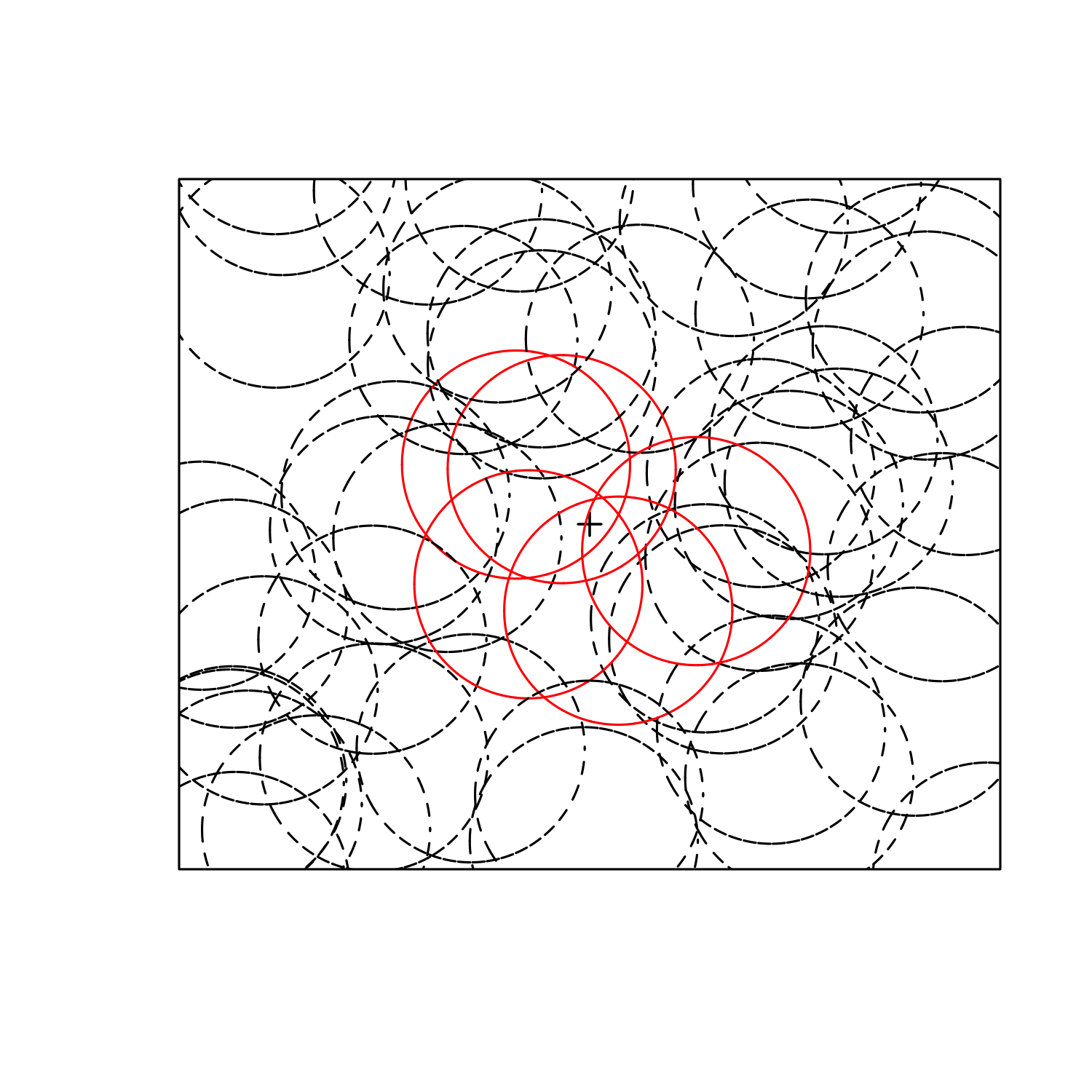


**Fig. S1 | An example of the distributions of species’ ranges modelled as disks.** The circles represent the species’ ranges and the cross shows the focal point, that is, the starting point of the sampling scheme. The circles highlighted in red indicate species whose ranges encompass the focal point.

Heaviside function is equal to zero for distances less than the closest individual’s distance to the focal point, and one for distances equal or greater than that of the closest point, being graphically a step. If the centre of the disk of species *i* is at distance *λ_i_* from the focal point, then the closest distance of the circle to the focal point is *λ_i_* - 2*σ_d_*, except for those species whose circles include the focal point. The latter are immediately detected (the red circles in Fig. S1). Let *S_c_* be the number of these species, which can be estimated based on the species density: $S_{c}=\pi{(2\sigma_{d})}^{2}{S_{T}}/{A_{T}}$. The SAR is, then, the summation of unit Heaviside step functions, *h*(*r-(λ_i_*-2*σ_d_*)),

$$S\left( r \right)=S_{c}+\sum_{i=S_{c}+1}^{S_{T}} h\left( r-\left( \lambda_{i}-2\sigma_{d} \right) \right). (S2.1)$$

The summation starts at *S_c_*+1 because there are *S_c_* species that contain the focal point.

If the probability mass function of the centroids, *λ*, is *p*(*λ*) with *K* classes, then the previous expression can be rewritten as

$$S\left( r \right)=S_{c}+S_{T}\sum_{j=1}^{K} p\left( {}_{j} \right)h\left( r-\left( \lambda_{j}-2\sigma_{d} \right) \right). (S2.2)$$

Converting the sum into an integral, and denoting the probability density function of *λ* by *f*(*λ*), it becomes

$$S\left( r \right)=S_{c}+S_{T}\int_{2\sigma_{d}}^{R_{T}} f\left( \right)h\left( r-\left( \lambda-2\sigma_{d} \right) \right)d. (S2.3)$$

The integral starts at 2*σ_d_* because only the species whose centroids are at a distance larger than 2*σ_d_* are relevant for the calculations. Since we assume that the centroids of the disks are uniformly randomly distributed, the distribution of the centroids is $f\left( \lambda\right)=2\lambda/R_{T}^{2}$. Re-writing the step function as $h\left( -\left( \lambda-(r+2\sigma_{d} \right) \right),$ the previous integral reduces to

$$S\left( r \right)=S_{c}+S_{T}\int_{2\sigma_{d}}^{r+2\sigma_{d}} \frac{2}{R^{2}}d, (S2.4)$$

from where we obtain

$$S\left( r \right)=S_{c}+S_{T}\left[ \frac{r^{2}+4r\sigma_{d}}{R^{2}} \right], (S2.5)$$

and, with $A=\pi r^{2}$ and $A_{T}=\pi R_{T}^{2}$, we finally obtain the SAR,

$$S\left( A \right)=S_{c}+S_{T}\left[ \frac{A+4\sqrt{\pi A}\sigma_{d}}{A_{T}} \right], (S2.6)$$

which is verified for $A\leq\pi\left( R-2\sigma_{d} \right)^{2}$. Expression S2.6 is equivalent to expression (2) in Allen and White^9^.

We present the outcomes of simulations conducted using this model in Fig. S2 with green dots, and plotted the curve corresponding to expression (S2.6) with a green line. Analogous to the simulations with bivariate normal distributions, for very large *A*, in a double logarithmic plot, the SAR tends toward an asymptote with slope 1. This result that can be retrieved from (S2.6) by observing that for large values of *A*

$$S\left( A \right)\simeq S_{c}+\frac{S_{T}}{A_{T}}A, (S2.7)$$

and, if $\frac{A}{A_{T}}S_{T}{\gg S}_{c}$,

$$S\left( A \right)\simeq\frac{S_{T}}{A_{T}}A. (S2.8)$$

For small area sizes, the number of species of the disk model increases very gradually because all *S_c_* species whose centres are at a distance smaller than 2*σ_d_* of the focal point are counted simultaneously. Consequently, for small areas, as *A* increases, few new species are recorded.


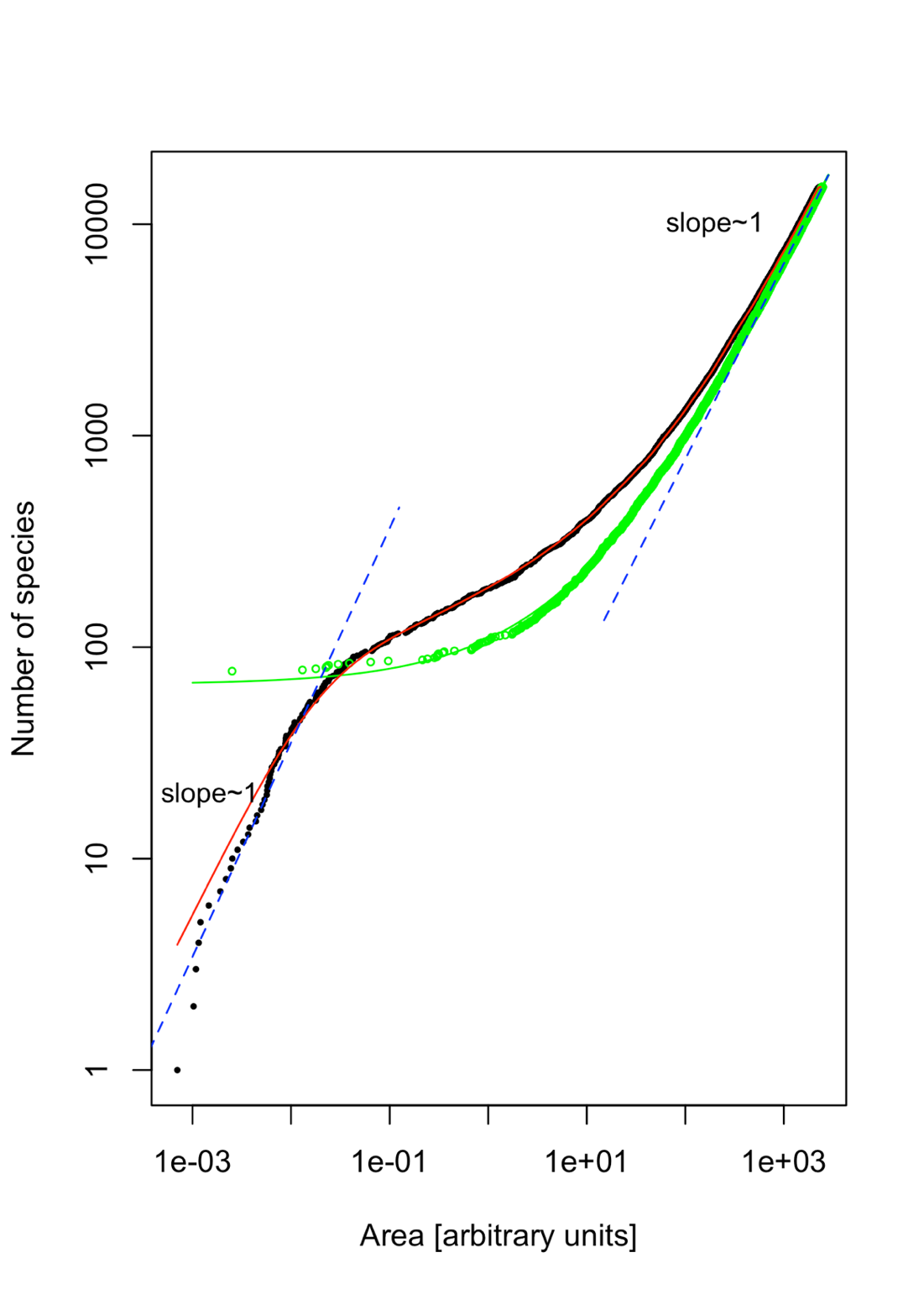


**Fig. S2 | The SAR obtained with simulations and the SAR predicted by the disk model.** The SAR depicted by black dots was obtained through simulations assuming 15,000 species, each with 1,000 individuals spatially distributed according to bivariate normal distributions with *σ*=1 and centres uniformly randomly distributed. The green points correspond to the SAR of the disk model, and the green line corresponds to the formula (S2.6). The red line was obtained with formula (1) from the main text. The dashed blue lines were obtained by fitting the SAR at very small and very large areas.

**Supplementary Note 3: The SAR and the evolution of parameters of the generalized extreme value (GEV) distribution**

The simulations detailed in the main text accurately reproduced the three phases of the SAR, as illustrated in Fig. 2a and S2. Examining the estimated parameters of the GEV distribution, $\hat{\mu}$, $\hat{\xi}$ and $\hat{\sigma}$, as a function of the corresponding location parameter, *υ_p_*, of the Rice (parent) distribution offers additional insights into the SAR’s shape (Fig. 3 of the main text). Recall that in the simulations the scale parameter of the bivariate normal distribution was set to *σ_n_* = 1, which corresponds to *σ_p_* = 1 of the Rice distribution (see Methods). Therefore, species featuring *υ_p_* < 2 are likely to encompass the focal point (see Fig. 4 of the main text). Within this range of *υ_p_*, the estimated GEV’s location parameter is $\hat{\mu}\cong0$, and the shape parameter is $\hat{\xi}\cong$ -0.5 (refer to Fig. 3 of the main text). This outcome suggests that species contributing to this region have the centres of their ranges in close proximity to the focal point, a zone where the Rice distribution is well approximated by a Rayleigh distribution. This observation was justified in Supplementary Note 1, where we use the Rayleigh distributions to obtain an approximation for the SAR for small areas of the form *S*=*cA*.

In the region 2 < *υ_p_* < 4 the three parameters of the GEV exhibit transitions between two distinct stages. Notably, the location parameter, $\hat{\mu}$, ceases to be close to zero and starts a gradual increase, eventually attaining a linear relationship for *υ_p_* > 4. This growth pattern is well approximated by a power law, as depicted in Fig. 3b, and can be related to the similarly approximated power law behaviour of the SAR in Phase II. To illustrate this connection, let’s assume that the cdf of the minima can be approximated by a step function, denoted as *h*, with a shift equal to the location parameter, *μ*. Thus, the expression

$$S\left( r \right)=\sum_{i=1}^{S_{T}} L\left( r;\mu_{i},\sigma_{i},\xi_{i} \right) (S3.1)$$

becomes

$$S\left( r \right)\simeq\sum_{i=1}^{S_{T}} h\left( r-\mu_{i} \right), (S3.2)$$

where, for simplicity, we start by working with distances, *r*. The step function approximation is deemed reasonable when the transition of the distributions of the minima from approximately zero to approximately 1 of the is small compared to the distances to the focal point, that is, when we observe a sharp transition. If the distribution of the location parameter is represented by *g*(*μ*) and there is a large number of species, the sum can be substituted with the integral

$$S\left( r \right)=S_{T}\int_{0}^{R_{T}} g(\mu)h\left( r-\mu\right)d\mu, (S3.3)$$

where *R_T_* is the radius corresponding to the total area. If species ranges are uniformly randomly distributed, then the distribution of *υ_p_* is *f*(*υ_p_*)=2*υ_p_*/$R_{T}^{2}$. Assuming a power-law relationship $\mu=\alpha\upsilon_{p}^{\beta}$, a transformation of variables leads to g$\left( \mu\right)=\frac{2}{R_{T}^{2}\beta\alpha^{2/\beta}}\mu^{\frac{2}{\beta}-1}.$ Substituting g$\left( \mu\right)$ in the above integral we obtain S$\left( r \right)=\frac{S_{T}}{{R_{T}^{2}\alpha}^{2/\beta}}r^{\frac{2}{\beta}}$, or in terms of area, $A=\pi r^{2}$, we get

$$S\left( A \right)=cA^{z}, (S3.4)$$

where $c=\frac{S_{T}}{\pi^{1/\beta}R_{T}^{2}\alpha^{2/\beta}}$ and $z=1/\beta$. From Fig. 3b we estimated *β* ≈ 3.45, consequently or *z* ≈ 0.29, a value that is not significantly different from the slope of 0.24 estimated from Fig. 2a.

Finally, for *υ_p_* > 4 the scale, $\hat{\sigma}$, and shape, $\hat{\xi}$, parameters estimates stabilize. The scale parameter remains approximately constant because for species far away from the focal point, only the location parameters of the Rice distribution and of the distribution of minima undergo changes, thus the scale parameter, *σ*, is approximately constant (see Fig. 3). More revealing, the shape parameter, $\hat{\xi}$, during this phase is approximately equal to 0, indicating a good approximation of the GEV distribution by a Gumbel distribution. The latter observation is expected because, when *υ_p_* $>$ 4*σ_p_*, the Rice distribution closely resembles a normal distribution, whose minima follow a Gumbel distribution^4^; see Fig. 4.

**Supplementary Note 4: Simulations**

In the main text, we assumed species spatial distributions (ranges) characterised by bivariate isotropic normal distributions, all sharing the same parameters and the same number of individuals, with their centres uniformly randomly distributed. Here, we relax some of these assumptions to reflect more realistic scenarios, which will help interpret the variety of SARs observed in the GBIF data. As before, we obtain a SAR by identifying, for each species, the individual closest to the focal point.

*The effect of range size*

We begin by illustrating in Fig. S3 the effect of range width, denoted by *σ*, on the shape of the SAR. Although, these results could also be interpreted as reflecting a situation where only a subset of the total area is observed, we present them here for completeness, as in some cases species, such as birds, may have very wide ranges relative to the total area. In these simulations, we assume that all species share the same *σ*. As *σ* increases, while keeping the total area constant, Phase III gradually diminishes and eventually disappears. In the latter case, the value of *S_P_*_(_*_II-III_*_)_ is equal to 1500 (not displayed in the figure), which corresponds to the total number of species assumed in the simulations, revealing that Phase III has not been reached. In agreement with our previous discussion, when *σ* increases, the number of estimated values of the shape parameter of the GEV distribution, $\hat{\xi}$, near zero decreases, while the occurrence of values closer to -0.5 increases; for the largest value of *σ* =8, $\hat{\xi}$ never reaches values near zero. SARs without Phase III resemble those observed for birds, for which Phase III is hardly observed (see Fig. 5 and Fig. S13).

**
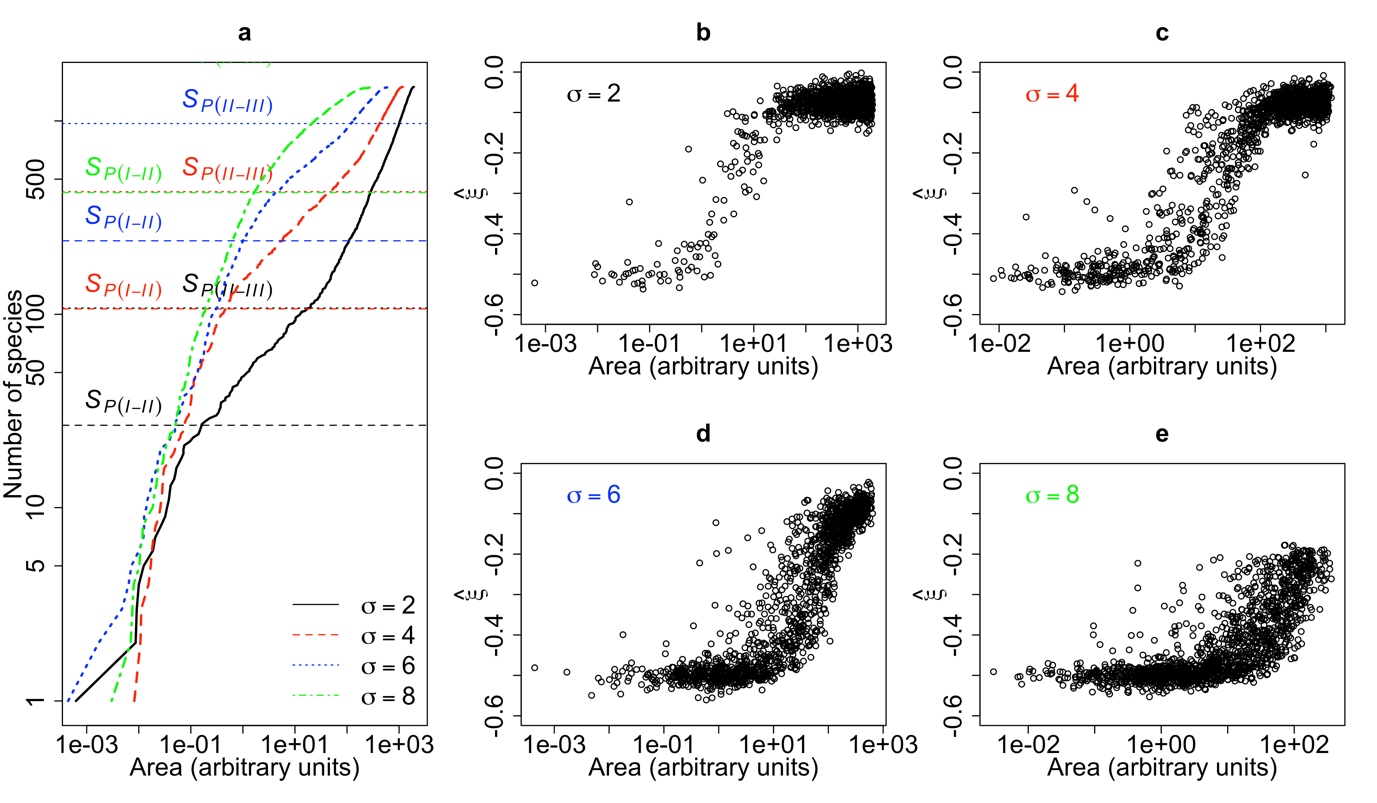
**

**Fig. S3 | The effect of the range size**. In each SAR shown in plot (**a**), species have ranges of equal width, specified by *σ*. As *σ* increases, Phase III progressively shrinks until it disappears entirely. Plots (**b-e**) show the transition of $\hat{\xi}$ as area increases for each of the four values of *σ* considered. The simulations were conducted with 1,500 species.

*Communities of species with varying range sizes*

In real communities, species exhibit different ranges. In Fig. S4, we show the results of simulations assuming species with range centres spatially distributed according to a uniform random distribution but with sizes, *σ*, that follow a normal distribution with a mean of 3 and standard deviations, sd(*σ*), of 0.3, 0.6, 0.9 and 1.2, but always ensuring that *σ*  only takes positive values (arbitrary units). In all cases, the SAR display a triphasic pattern, see Fig. S4a. Although some variability is expected due to the randomness inherent in the simulations, for those species contributing to Phases II and III, we expect that communities with larger sd(*σ*) will have SARs with more species for the same area. This occurs because, for species with ranges centres located at approximately at the same distance from the focal point, those with larger ranges will have individuals closer to the focal point and, thus, are detected earlier. For increasing values of sd(*σ*), we observed the following values of *S_P_*_(_*_I-II_*_)_ = 415, 421, 444, 466 and *S_P_*_(_*_II-III_*_)_= 1609, 1671, 1748, 1863.

**
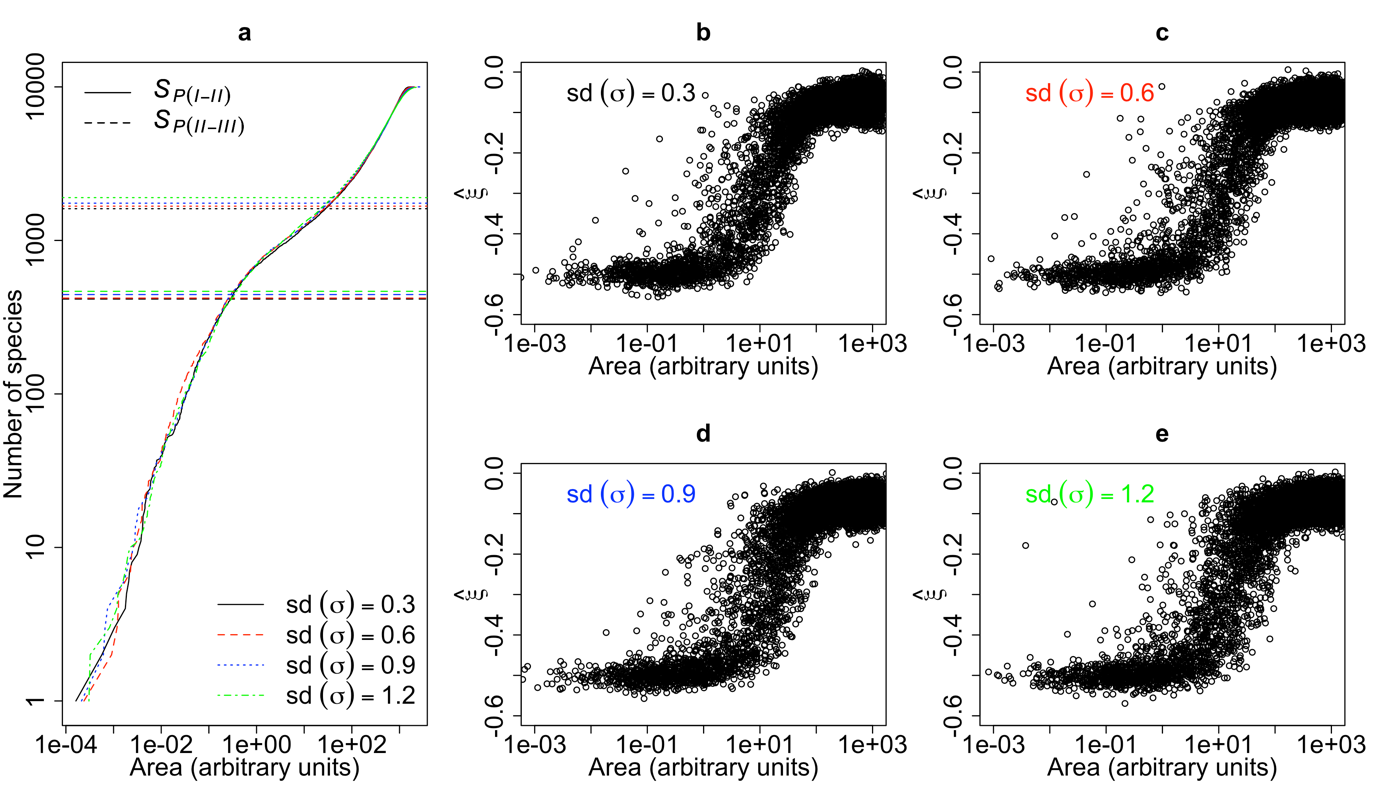
**

**Fig. S4 | Communities with species with different range sizes.** The SARs in plot (**a**) correspond to species with range sizes, *σ*, sample from a normal distribution with a mean of 3 and standard deviation (sd) of 0.3, 0.6, 0.9 and 1.2, black, red, blue and green curves, respectively (arbitrary units). Plots (**b-e**) show the transition of $\hat{\xi}$ as area increases for each of the four values of sd(*σ*) considered. The simulations were conducted with 10,000 species.

*The SAR of species with ranges centres spatially distributed according to bivariate isotropic normal distributions*

In real-world scenarios, the centres of species’ ranges are not spatially uniformly randomly distributed, and communities are likely to exhibit spatial gradients in species richness. Here, we simulate SARs from species whose range centres are spatially distributed according to an isotropic bivariate normal distribution. We first consider normal distributions centred at the origin, but with different standard deviations: 20 and 30 (arbitrary units). Otherwise, the species are characterised as in the simulations of Fig. 2 of the main text. The results are displayed in Fig. S5. In both cases, the SARs exhibit a triphasic pattern, plot (**a**). As expected, the SAR for the distribution with smaller standard deviation shows more species for the same area (indicating a higher species density). Only at very large areas does the SAR for the distribution with larger standard deviation reaches the same number of species (since the number of species is equal in both simulations). The slope values across the three phases follow the same trend as before: steep in Phase I, decreasing in Phase II and rising again in Phase III. Plots (**b-c**) show the transition of $\hat{\xi}$ as area increases.


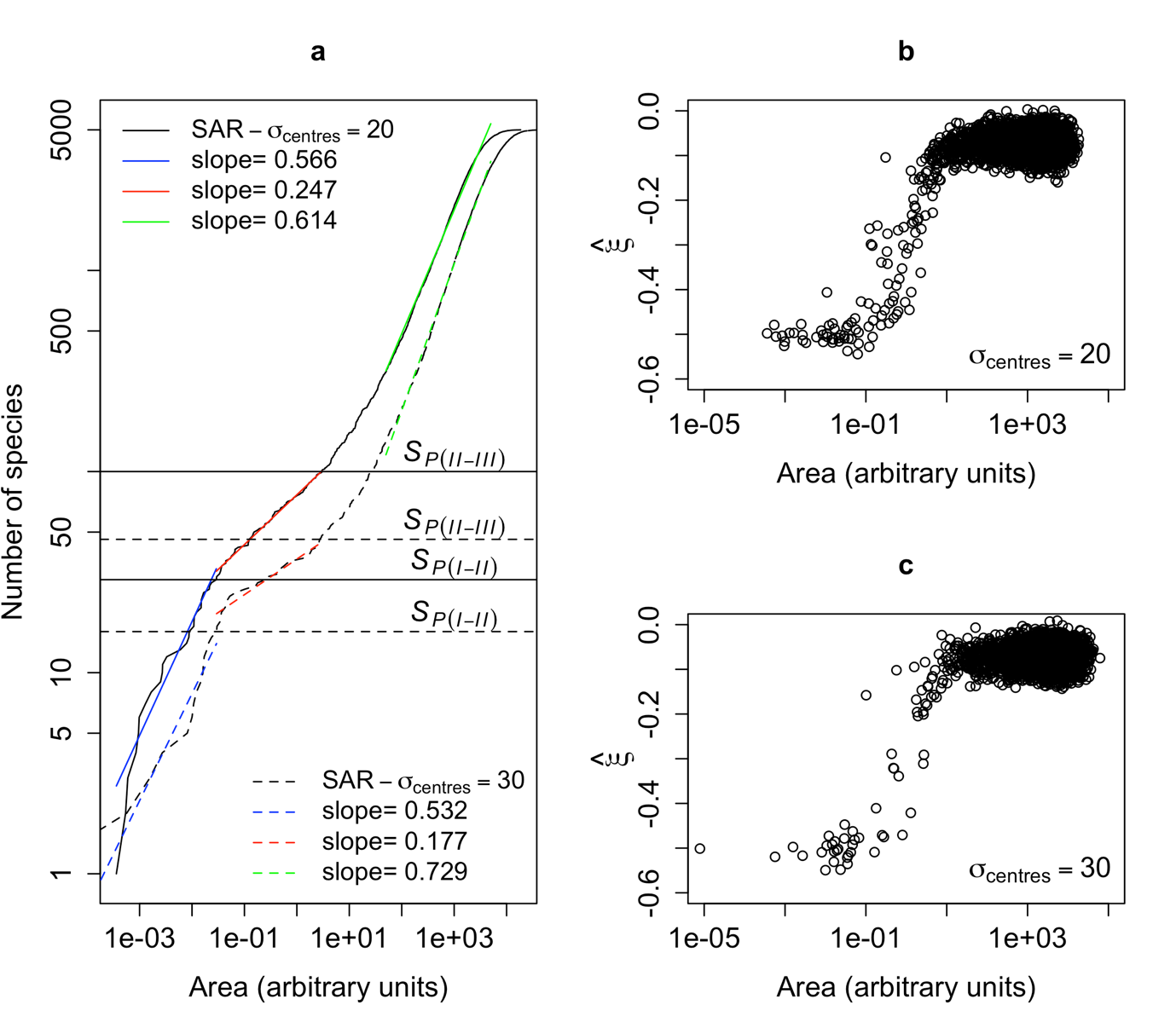


**Fig. S5 | SARs of communities with species ranges distributed according to an isotropic bivariate normal distribution centred at the origin.** In plot (**a**) the above SAR was obtained assuming that the isotropic bivariate normal distribution had a standard deviation of 20, while the SAR below was obtained with a standard deviation of 30 (arbitrary units). Plots (**b-c**) show the corresponding transitions of the $\hat{\xi}$ as area increases. Note that the transition between phases occurs at approximately the same area sizes.

Additionally, we carried simulations assuming that the distributions of the range centres are not centred on the focal point. Figure S6 shows the results for two distributions: one centred at a distance of 15 units the focal point, and another at a distance of 30. In both cases the standard deviation was 20 (arbitrary units). As expected, the SAR of the community with range centres closer to the focal point shows more species for the same area compared to the community with centres farther away. The observations made regarding the SARs in Fig. S5 also apply here.


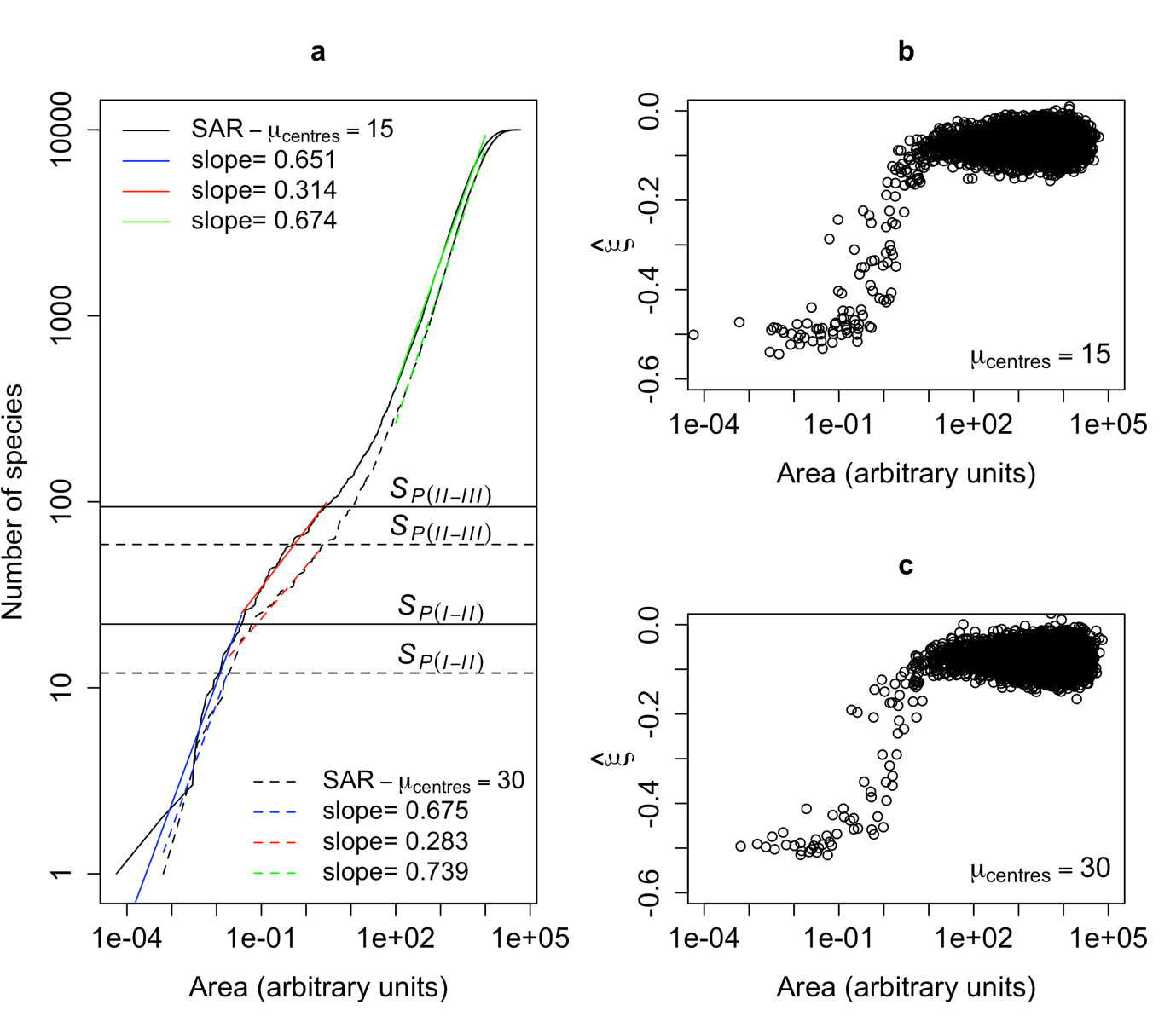


**Fig. S6 | SARs of communities with species ranges distributed according to an isotropic bivariate normal distribution not centred at the origin.** In plot (**a**) the upper SAR was obtained with range centres following an isotropic bivariate normal distribution having standard deviation of 20, while lower SAR was obtained with a standard deviation of 30 (arbitrary units). Plots (**b-c**) show the corresponding transitions of the $\hat{\xi}$ as area increases.

To conclude this section, we consider two extreme cases of the previous simulations: one with the centre of the bivariate normal distribution at a distance of 30 units and another at a distance of 45 units, both with standard deviation equal to 10. This choice of values ensures that most species’ ranges do not include the focal point. Based on our earlier discussions, this implies that the SARs should have only Phase III, or, at the most, a Phase II with a small number of species. Indeed, this is what we observe in Fig. S7a, as well as in the values of $\hat{\xi}$ shown in Fig. S7b,c. Furthermore, the slope of Phase III is larger than 1, indicating that the rate at which new species are found increases as the sampled area expands. This is possible because we initially detect only a few species at the edge of the bivariate normal distribution of the range centres, but as the sampling area expands towards the centre of the distribution, more species are observed.

The latter simulations are important to interpret some of the SARs observed in the GBIF data. For instance, like the SARs in Fig. S7, the SAR derived from GBIF data for amphibians in North America, does not exhibit Phase I; see Figure S13i below. To understand the reasons for pattern exhibit by this SAR, we present in Fig. S8 the locations of the focal point, of the data occurrences, and the of occurrences corresponding to the minimum distance to the focal point for the 94 species included in this SAR. A closer examination reveals that most occurrences, as well as the minima, are situated predominantly south of the focal point, which suggests that it is unlikely that the species’ ranges include the focal point; as in the simulations that led to Fig. S7. Therefore, as discussed previously, we expect that few species are likely to contribute to Phase I, and only a small number will be part of Phase II. Indeed, we obtained *S_P_*_(_*_I-II_*_)_=1 and *S_P_*_(_*_II-III_*_)_=6.

**
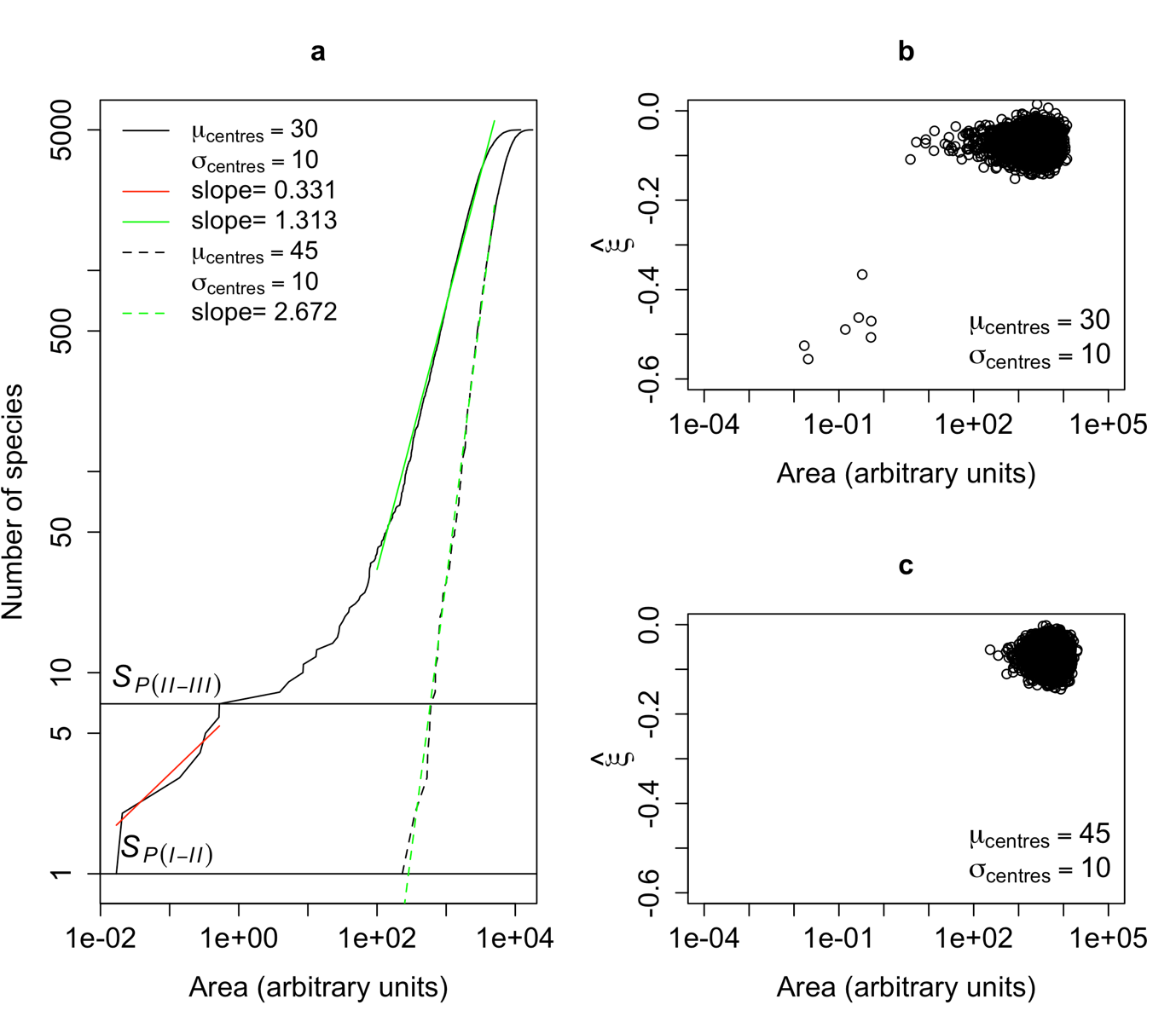
**

**Fig. S7 | SARs of communities with species ranges distributed according to an isotropic bivariate normal distribution centred far from the focal point.** In plot (**a**), the upper SAR was obtained assuming that an isotropic bivariate normal distribution of the range centres was centred 30 units from the focal point, while the lower SAR corresponds to a distribution centred 45 units away. Both distributions have a standard deviation of 10. Plots (**b-c**) show the corresponding transitions of $\hat{\xi}$ as area increases. Note the steep slopes of the SARs for Phase III and the values of $\hat{\xi}$ approaching zero (as expected for Phase III).

**
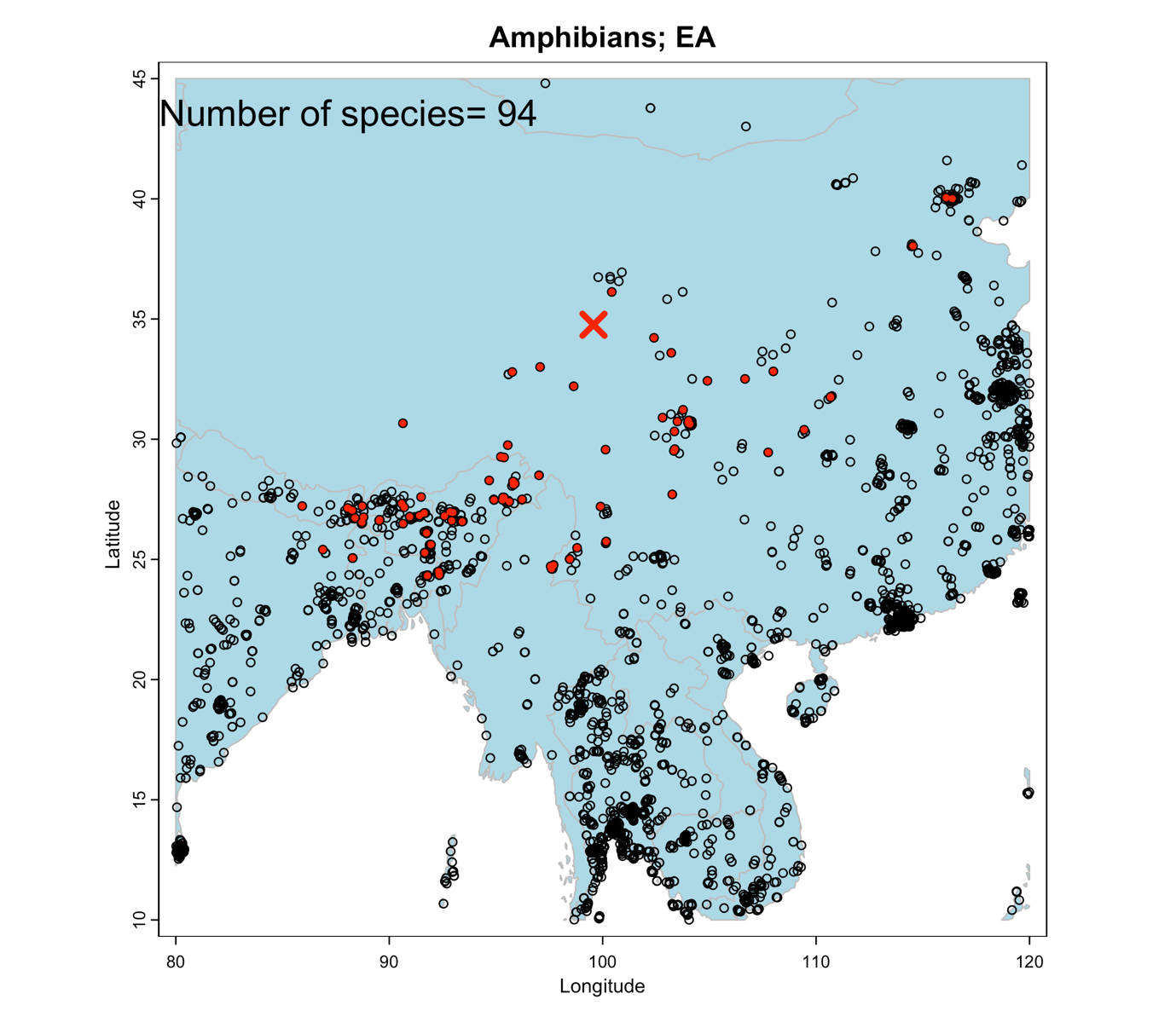
**

**Fig. S8 | The location of the focal point, of the records and of the minima that led to the SAR of amphibians in Eurasia, Fig. S13i.** Although the locations of all the records corresponding to the 94 species contributing to this SAR are present in other parts of Eurasia (EA), we zoom in the map to highlight the region where the minima occur. Most GBIF occurrences, as their corresponding minima distances to the focal point, are located south of the focal point, revealing that it is unlikely that the range of the species include the focal point. In fact, we estimated *S_P_*_(_*_I-II_*_)_=1 and *S_P_*_(_*_II-III_*_)_=6. Map generated using data from Natural Earth (http://www.naturalearthdata.com).

*Evaluating the impact of species abundance variability*

Thus far, we have not explicitly considered species abundances, as measured, for example, by the number of individuals. Abundance has been implicitly accounted for in the range sizes, as we might assume that more abundant species have larger ranges. However, this is not necessarily the case (see Fig. S9). Here, we explore what happens when we consider abundances explicitly. Nevertheless, we argue that, when determining the SAR, the most critical feature is the range size, as this is the characteristic most likely to influence the proximity of the individual closest to the focal point. Therefore, we also performed simulations for communities with the same distribution of range sizes, but assuming that all species have the same number of individuals.

The relationship between range size and abundance is complex, therefore, instead of establishing this relationship a priori, we used the one observed for reptiles in North America, Fig. S9; the relationship is similar for other taxa. To obtain a SAR, we sampled with replacement the data on range size and the corresponding abundances, the required number of species, and then assumed isotropic bivariate normal distributions with centres uniformly randomly distributed. Simultaneously, we performed simulations for the same number of species with the same range sizes, but assuming all species have the same abundance. The results are presented in Fig. S10 where, as before, the SARs exhibit a triphasic pattern. As expected, the SARs obtained from both approaches are similar, with the curves effectively superimposed. We recognised that this is a simple model and that further research is warranted with more realistic models, but those are beyond the scope of this work.


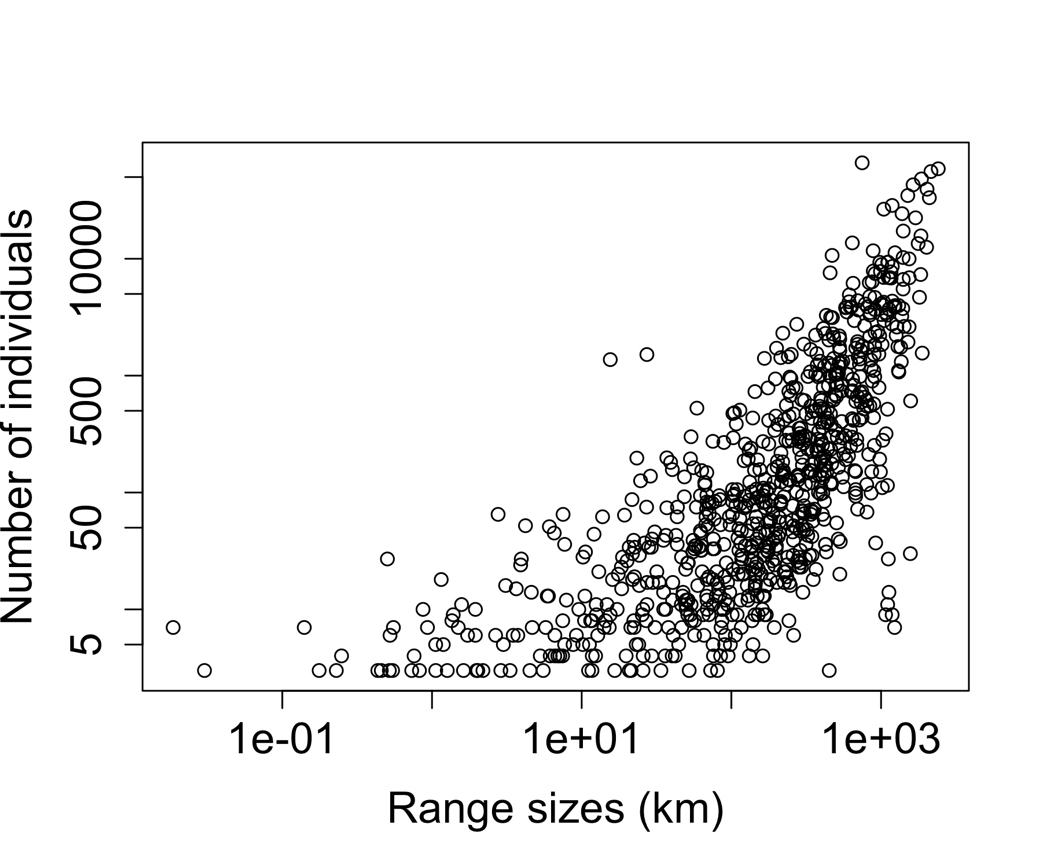


**Fig. S9. The relationship between range size and abundance.** This figure was obtained using the data on reptiles in North America.

**
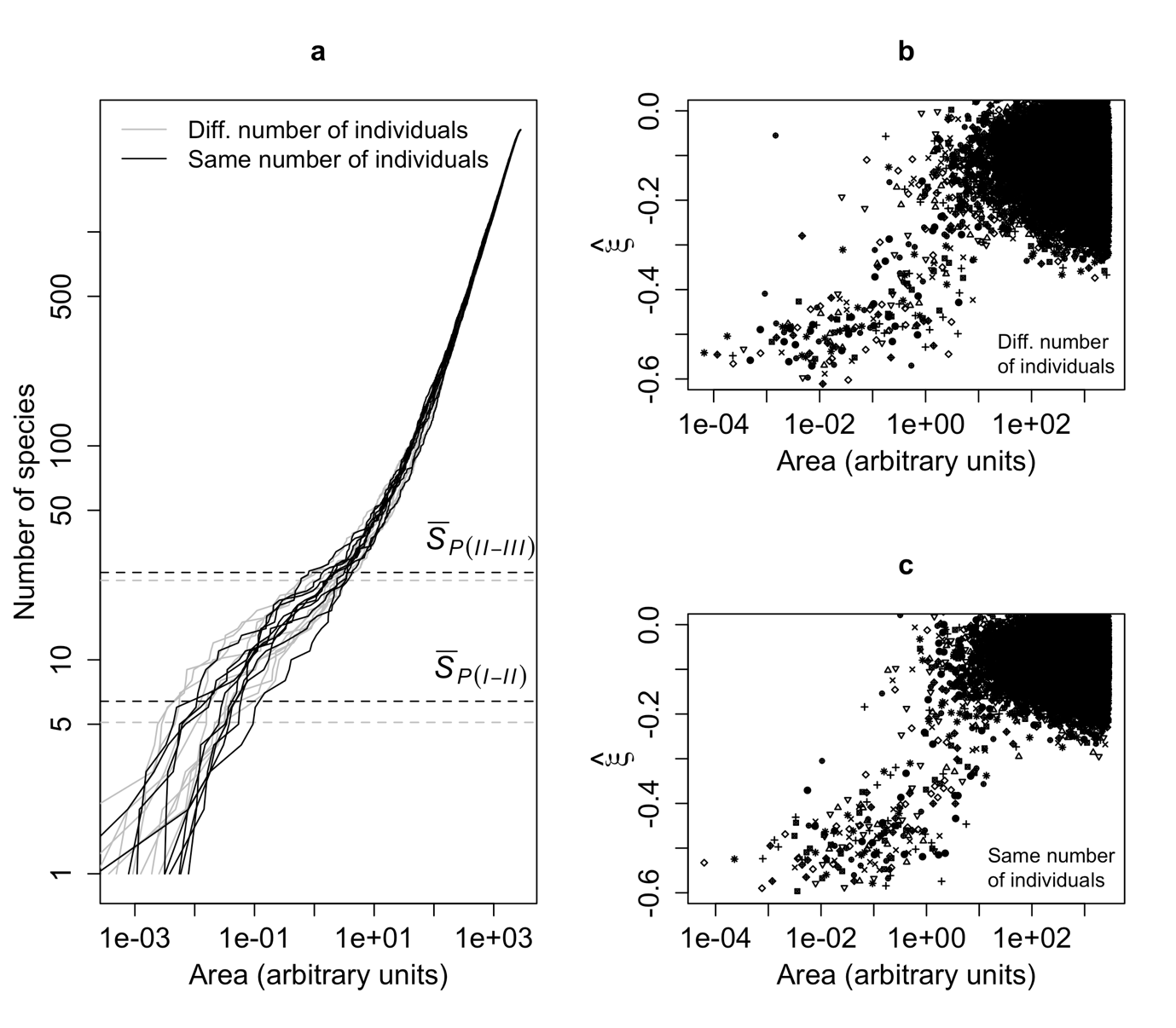
**

**Fig. S10| SARs obtained from species with different and equal number of individuals.** In plot (**a**), the ten grey lines correspond to the SARs obtained from the relationship between range size and abundance as for reptiles in North America, Fig. S9, and the ten black lines depict SARs obtained for the same range sizes but with species having an equal number of individual The values of $\bar{S}_{P(I-II)}$ and $\bar{S}_{P(II-III)}$ are the mean values obtained from the SARs. Plot (**b**) show the transitions of $\hat{\xi}$ as area increases for the case of species having different number of individuals and plot (**c**) represents the transitions for the scenario of species with equal number of individuals.

*The SAR of species with ranges given by bivariate isotropic Cauchy distributions*

The simulations in the main text, and the previous ones in this section, assumed species with ranges characterized by bivariate isotropic normal distributions, which have “thin” tails. In reality, communities are likely to consist of a combination of species with both “thin” and “fat” tailed distributions. Here, to illustrate the consequences of fat tails, we analyse the SAR resulting from species with ranges defined by isotropic bivariate Cauchy distributions.

The probability density function of the bivariate Cauchy distribution is given by

$$f\left( x,y;\gamma,x_{0},y_{0} \right)=\frac{1}{2\pi}\left[ \frac{\gamma}{\left[ \left( x-x_{0} \right)^{2}+\left( y-y_{0} \right)^{2}+\gamma^{2} \right]^{\frac{3}{2}}} \right], (S4.1)$$

where *x*_0_ and *y*_0_ are the coordinates of the centre of the distribution and *γ* is a scale parameter (*53*). In the case of distributions centred on the origin, *x*_0_ = *y*_0_ = 0, expression S4.1 simplifies to

$$f\left( x,y;\gamma\right)=\frac{1}{2\pi}\left[ \frac{\gamma}{\left( {\gamma^{2}+x}^{2}+y^{2} \right)^{\frac{3}{2}}} \right]. (S4.2)$$

To derive the distribution of distances, we utilize the transformation of variables *x*=*r*cos(*θ*) and *y*=*r*sin(*θ*), where *θ* represents the angle whose Jacobian is *r*. Upon integrating over *θ*, the previous expression becomes

$$f\left( r;\gamma\right)=\frac{\gamma r}{\left( {\gamma^{2}+r}^{2} \right)^{3/2}} (S4.3)$$

with the corresponding cdf being

$$F\left( r;\gamma\right)=\int_{0}^{r} \frac{\gamma x}{\left( {\gamma^{2}+x}^{2} \right)^{3/2}}dx=1-\frac{\gamma}{\left( {r^{2}+\gamma}^{2} \right)^{1/2}}. (S4.4)$$

This distribution of distances for the bivariate isotropic Cauchy distribution is equivalent to the Rayleigh distribution of distances for the bivariate normal distribution centred on the origin. For *r*>>*γ* the probability density function (pdf) can be approximated by

$$f\left( r;\gamma\right)\approx\gamma r^{-2}, (S4.5)$$

that is, a power law with exponent -2, precisely mirroring the characteristics of the Cauchy distribution. As a side note, *f*(*r*;*γ*) does not have mean (and consequently variance) due to the divergence of the integral $\int_{0}^{\infty} rf\left( r,\gamma\right)dr$. This divergence is expected, considering its association with the Cauchy distribution.

For bivariate Cauchy distributions centred on the origin, the asymptotic distribution of the minima can be obtained, as in Supplementary Note 1, by introducing a linear normalization, *r*= *a_n_* *x* + *b_n_*. The cdf of the minima, *L*(*x*;*γ*), becomes

$$L\left( x;\gamma\right)=\lim_{n\to\infty} \left( 1-\left[ \frac{\gamma}{\sqrt{\gamma^{2}+\left( a_{n}x+b_{n} \right)^{2}}} \right]^{n} \right), (S4.6)$$

and assuming $a_{n}=\sqrt{2/n}$ and $b_{n}=0$ (as previously employed for the Rayleigh distribution), we obtain

$$L\left( x;\gamma\right)=\lim_{n\to\infty} \left( 1-\left[ \frac{1}{\sqrt{1+\frac{x^{2}}{\gamma^{2}n/2}}} \right]^{n} \right)=\lim_{n\to\infty} \left( 1-\left[ \frac{1}{\left( 1+\frac{x^{2}}{\gamma^{2}n/2} \right)^{n/2}} \right] \right), (S4.7)$$

Or

$$L\left( x;\gamma\right)=1-\exp\left( -\frac{x^{2}}{\gamma^{2}} \right). (S4.8)$$

Similar to the distribution derived for the minima of the Rayleigh distribution, S4.8 corresponds to a Weibull distribution with scale parameter, *γ*, and shape parameter equal to 2 (or a GEV with shape parameter *ξ* =-0.5). Therefore, as for the Rayleigh, we will obtain for small *r*, *S* ∝ *A*.

For completeness, we derive the analogue of the Rice distribution to the isotropic bivariate Cauchy distribution, expression S4.1. Employing a procedure similar to that used to obtain the Rice distribution^8^, we perform a change of variables with *x*=*r*cos(*θ*) and *y*=*r*sin(*θ*), whose Jacobian is *r*. Additionally, we introduce new constants *μ* and *ψ* defined as $x_{0}=\mu cos(\psi)$ and $y_{0}=\mu sin(\psi)$. Following these transformations (S4.1) simplifies, after some algebraic manipulations, to

$$f\left( r,\theta;\gamma,\mu,\psi\right)=\frac{1}{2\pi}\left[ \frac{r\gamma}{\left[ r^{2}+\mu^{2}+\gamma^{2}-2r\mu\cos(\theta-\psi) \right]^{\frac{3}{2}}} \right]. (S4.9)$$

Introducing $\phi=\theta-\psi$ , and considering the periodicity of the cosine, we obtain

$$f\left( r;\gamma,\mu\right)=\frac{r\gamma}{2\pi}\int_{0}^{2\pi} \frac{1}{\left[ a-bcos(\phi) \right]^{\frac{3}{2}}}d\phi, (S4.10)$$

where $a=r^{2}+\mu^{2}+\gamma^{2}$ and $b=2r\mu$. The solution of the previous integral is

$$f\left( r;\gamma,\mu\right)=\frac{r\gamma}{\pi}\left[ \frac{\prod\left( \frac{2b}{b-a};\pi|\frac{2b}{b-a} \right)}{\left( a-b \right)^{\frac{3}{2}}} \right], (S4.11)$$

where $\prod\left( n;z | m \right)$is an incomplete elliptic integral of the third kind^10^. Unfortunately, Equation (S4.11) is complicated and does not readily lend itself to simple analytical simplifications. We intend to explore it in future work.

Figure S11 shows a SAR obtained with simulations using the bivariate isotropic Cauchy distribution, and the correspondent evolution of the shape parameter *ξ* of the *GEV_m_*. Similar to previous observations, and according to our previous results, we observe a slope of 1 for Phases I and III. The estimated shape parameter, $\hat{\xi}$, is approximately equal to -0.5 for Phase I. However, an important difference from Fig. 3c is that now $\hat{\xi}$ tends to 1 as the area increases, whereas previously it tended to 0. This new finding reveals that the distributions of the minima for distributions very far from the focal point follow now Fréchet distributions. This was expected, given that the distribution of the minima of the Cauchy distribution is a Fréchet distribution^4^.


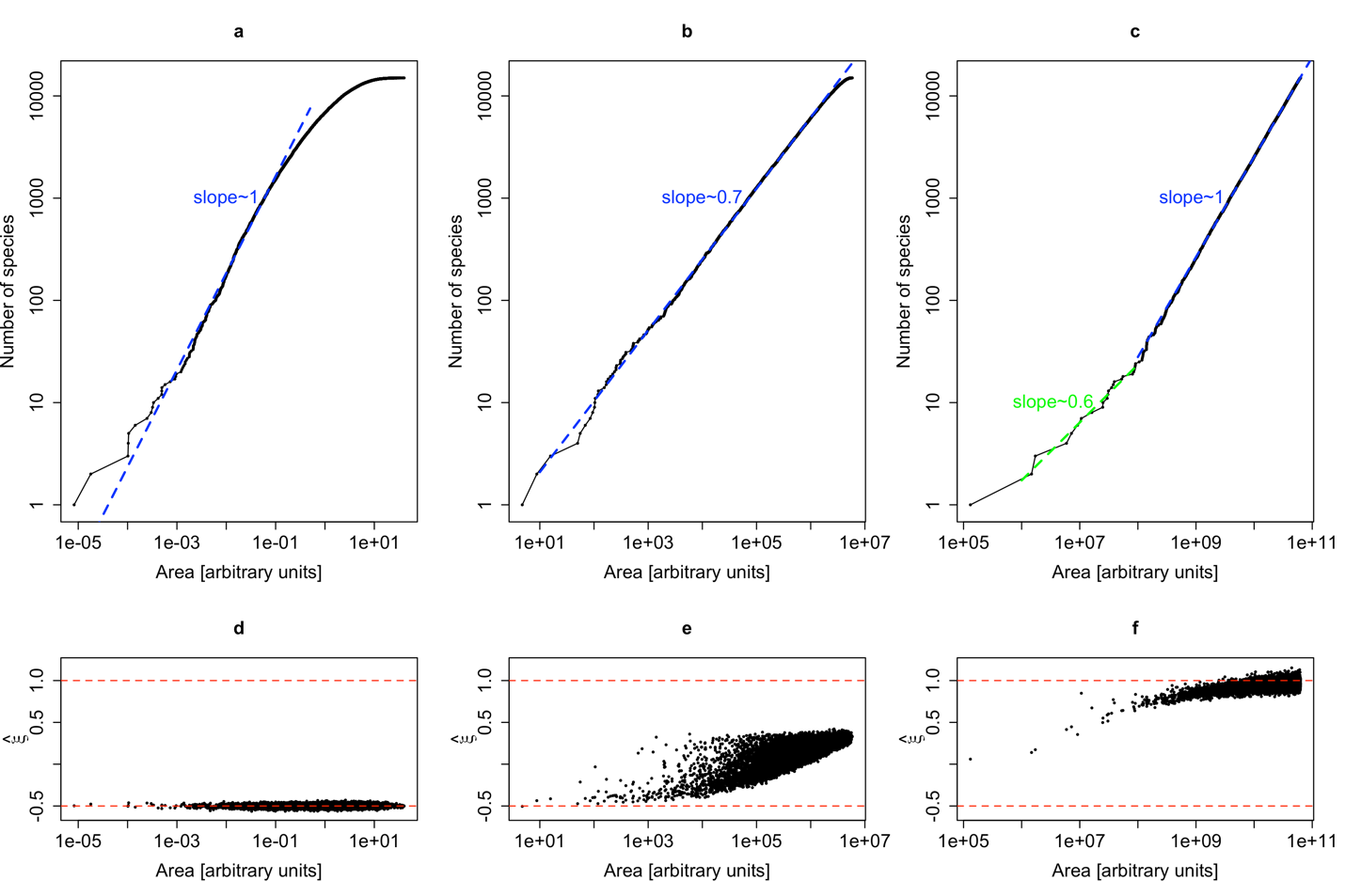


**Fig. S11 | The SAR obtained with isotropic bivariate Cauchy distributions (expression S4.1).** This figure mirrors Fig. 2 of the main text. However, due to the wide range of area sizes (expected when dealing with fat-tailed distributions), we display the three different phases in three plots: plot (**a**) corresponds to Phase I, plot (**b**) to Phase II, and plot (**c**) to Phase III. Plots (**d**), (**e**) and (**f**) illustrate the shape parameter of the GEV distribution. The notable difference from the results presented in Figs. 2 and 3 is that now *ξ* tends to 1 in Phase III, indicating a Fréchet distribution.

**Supplementary Note 5: The number of species at the transition between phases when the distributions of points are uniformly randomly distributed**

In the main text we showed that the number of species at the transition, *S_Pw_* where *w*=2*σ* or *w*=4*σ* , corresponding to *S_P_*_(_*_I-II_*_)_ and *S_P_*_(_*_II-III_*_)_, respectively, is given by

$$S_{Pw}=S_{T}\int_{0}^{R_{T}} \int_{0}^{2\pi} \mathbb{P}\left( w>r | r,\phi\right)f_{\upsilon_{p}}\left( r,\phi\right)d\phi dr. (S5.1)$$

where *S_T_* is the total number of species, $f_{\upsilon_{p}}\left( r,\phi\right)$ is the probability density function of the distribution of the ranges’ centres, and $\mathbb{P}\left( w>r | r,\phi\right)$ is the probability that a species has *w*>*r* given that it is present at $\left( r,\phi\right)$.

The above integral can be simplified if we assume isotropy and that the size of a range is independent on the location of its centre, i.e., $\mathbb{P}\left( w>r | r,\phi\right)\mathbb{=P(}w>r)$. Furthermore, if we denote the cdf of the sizes *w* by $F_{W}(r)$, then $\mathbb{P}\left( w>r \right)=1-F_{w}\left( r \right)$, and the previous integral becomes

$$S_{Pw}=S_{T}\int_{0}^{R_{T}} (1-F_{W}(r))f_{\upsilon_{p}}\left( r \right)dr. (S5.2)$$

If the ranges’ centres are uniformly randomly distributed than $f_{\upsilon_{p}}(r)=2r/R_{T}^{2}$, and using a transformation of variables $A=\pi r^{2}$, we obtain

$$S_{Pw}=S_{T}\int_{0}^{A_{max}} (1-F_{W}(A))\frac{2}{R_{T}^{2}}\sqrt{\frac{A}{\pi}}\frac{1}{2\sqrt{\pi A}} dA, (S5.3)$$

where $A_{max}=\pi R_{T}^{2}$, and

$$S_{P}=\frac{S_{T}}{A_{max}}\int_{0}^{A_{max}} (1-F_{W}(A)) dA. (S5.4)$$

Notice that $\left( 1-F_{W}\left( A \right) \right)$ is a survival function, and since

$$\int_{0}^{A_{max}} \left( 1-F_{W}\left( A \right) \right)dA=\bar{A}, (S5.5)$$

where $\bar{A}$ denotes the average area, we obtain

$$S_{P}=\frac{\bar{A}}{A_{max}}S_{T}, (S5.6)$$

a result we used to obtain to *S_P_*_(_*_I-II_*_)_ and *S_P_*_(_*_II-III_*_)_ from the simulations.

*An important property of S_P_*_(_*_I-II_*_)_ *and* *S_P_*_(_*_II-III_*_)_

Notice that species contribute to the SAR in a sequential manner. Specifically, species contributing to Phase I have their nearest individual to the focal point at a shorter distance (minimum) smaller than those contributing to Phase II and these at a shorter distance than those contributing to Phase III. However, our method of assigning species to different phases may result in instances that contradict the previous observation. This discrepancy can be illustrated using the simulations used the main text, i.e., even when all species have the same range sizes (same *σ*); see Fig. S12. Consider, for instance, Phases I and II. According to our criteria, species for which the distance from the centre of their range to the focal point, *d_FP_*, exceeds 2*σ* do not contribute to Phase I. However, as depicted in Fig. S12b, some species with *d_FP_* > 2*σ* have minimum values falling within the range defined as Phase I (the horizontal red line). The same applies for the species that identify Phases I and III, Fig. S12a and S12c. This happens because the location of the minimum distance in relation to the focal point of a species whose range centre is at a distance *d_FP_*_1_, may be smaller than that of a species whose range’s centre distance to the focal point, *d_FP_*_2_, is smaller than *d_FP_*_1_. In other words, the correlation between the location of the minima relatively to the focal point and the distance of the range’s centres is not perfect.


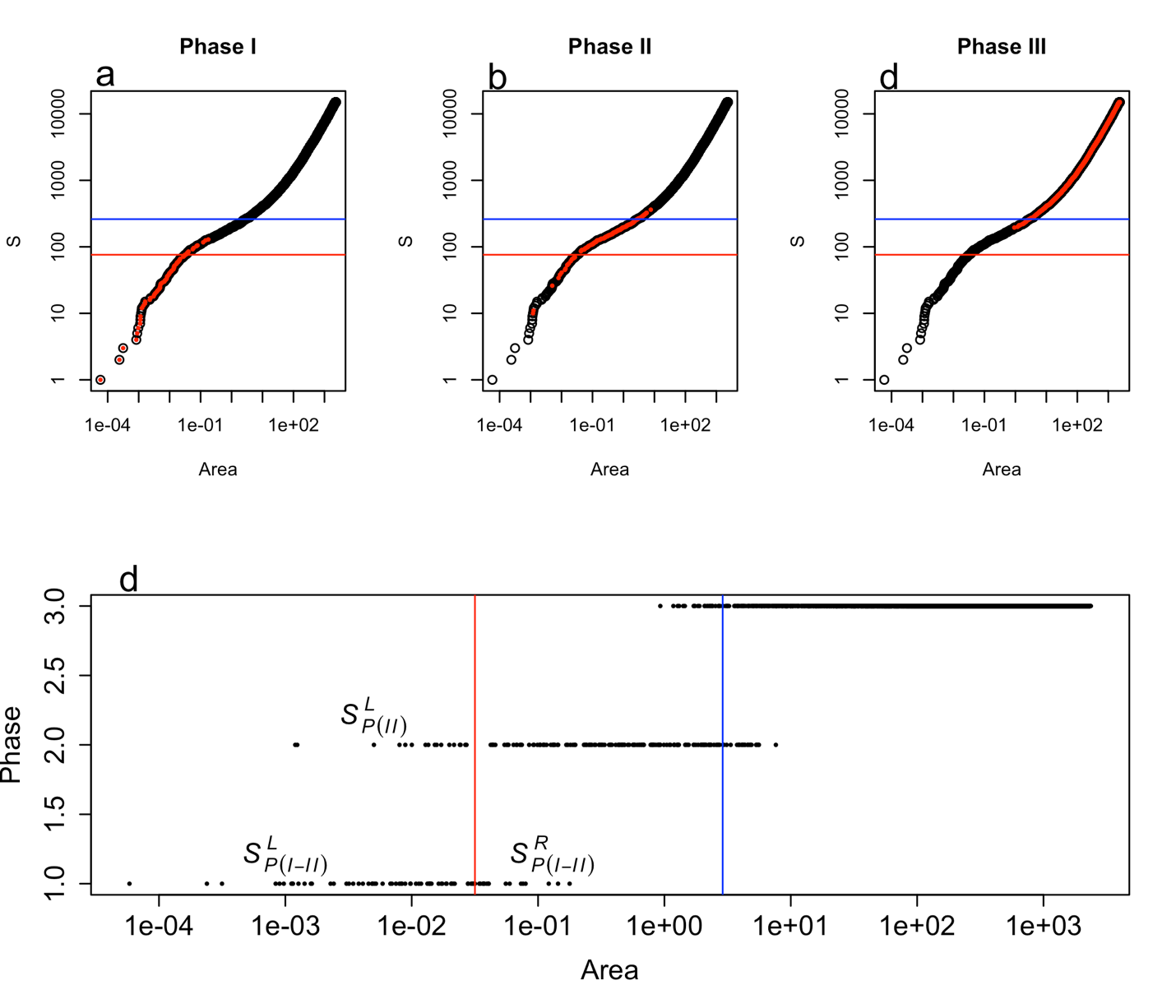


**Fig. S12 | The location of the minima and their contribution to the different phases. a**,**b**,**c**, The open dots correspond to the SAR and the red dots identify the location in the SAR of the minima as identified by the criteria: Phase I - *d_FP_* ≤ 2*σ* (**a**); Phase II 2*σ* < *d_FP_* ≤ 4*σ* (**b**); and Phase III *d_FP_* >4*σ* (**c**). The horizontal red and blue lines in these plots correspond to $S_{P(I-II)}$ and $S_{P(II-III)}$, respectively. **d**, The phases to which each minimum contribute to the SAR according to the previous criteria. The vertical red and blue lines identify the areas corresponding to $S_{P(I-II)}$ and $S_{P(II-III)}$, respectively.

The above observation may raise doubts about the accuracy of the criteria proposed to identify the transitions among phases. However, as we now show, this concern is unfounded. Consider Fig. S12d where we show the area corresponding to the position of the minima and the phases to which they contribute, according to our criteria. Without loss of generality, consider only the transition between Phases I and II. The vertical red line in Fig. S12d identifies the area corresponding to $S_{P(I-II)}$ species in the SAR (obtained with our criteria). Decompose $S_{P(I-II)}$ into the number of species to the left (*L*) and to the right (*R*) of this line, that is,

$S_{P(I-II)}$=$S_{P\left( I-II \right)}^{L}$+$S_{P\left( I-II \right)}^{R}. (S5.7)$

On the other hand, the total number of species identified as belong to Phase II according to our criteria, but that are in fact to the left of the red line, that is, in Phase I, is $S_{P\left( II \right)}^{L}$. And we know that

$S_{P(I-II)}$=$S_{P\left( I-II \right)}^{L}$+$S_{P\left( II \right)}^{L}, (S5.8)$

therefore, comparing the two previous expressions, we conclude that

$S_{P\left( I-II \right)+}$=$S_{P(II)-}$,$(S5.9)$

that is, the number of species identified as belong to Phase I but whose minima contribute to Phase II is exactly the same as the number of species identified as belong to Phase II but whose minima contribute to Phase I. Thus, $S_{P(I-II)}$ obtained with our criteria, encompasses all the species that indeed contribute to Phase I. The same reasoning applies to the transition between Phases II and III.

**Supplementary Note 6: The species-area relationships for amphibians, birds, mammals and reptiles in Africa, Australia, Eurasia, North-America and South-America**

Here we provide the SARs for amphibians, birds, mammals and reptiles in Africa, Australia, Eurasia, North-America and South-America. Figure S13 is the equivalent to Fig. 5 of the main text (and includes its plots). Tables S2 and S3 contain further information on the values of *S_P_*_(_*_I_*_-_*_II_*_)_ and *S_P_*_(_*_II_*_-_*_III_*_)_, and the values of the slopes associated with each phase.

Based on Fig. S13 and table S3, we observe that the slope of Phase II is smaller than those of the other two phases, except for amphibians in South America. In fact, both amphibians and birds in South America show a rapid increase in some regions of the “mean SARs”, Fig. S13q and S13r. This occurs because several species are registered in the same location, which leads to artificial high slopes; an issue we have highlighted in the main text. We hope this problem will be solved in the future as more data is collected and their locations more precisely recorded.

In the case of the amphibians in Eurasia we do not observe Phase I, Fig. S13i. The focal point of this “mean SAR” is located at (34.8ºN, 99.6ºE), approximately in the centre of People’s Republic of China. Interestingly, of the 94 amphibian species in this SAR, 24 species are represented by only a single individual. This suggested that there will likely be a considerable increase in the number of specimen in the future for this geographical region.

Figure S14 shows the histograms of the width of the distributions of distances of the individuals to the centre of the ranges for all taxa and landmasses, information that that was used to choose the taxa and landmasses to present in Fig. 5 of the main text. We fitted these histograms with a normal distribution. Although a basic test (Shapiro-Wilk test) revealed that none of the empirical distributions is well modelled by a normal distribution, visual assessment reveals that none of the distribution exhibits fat tails.

**
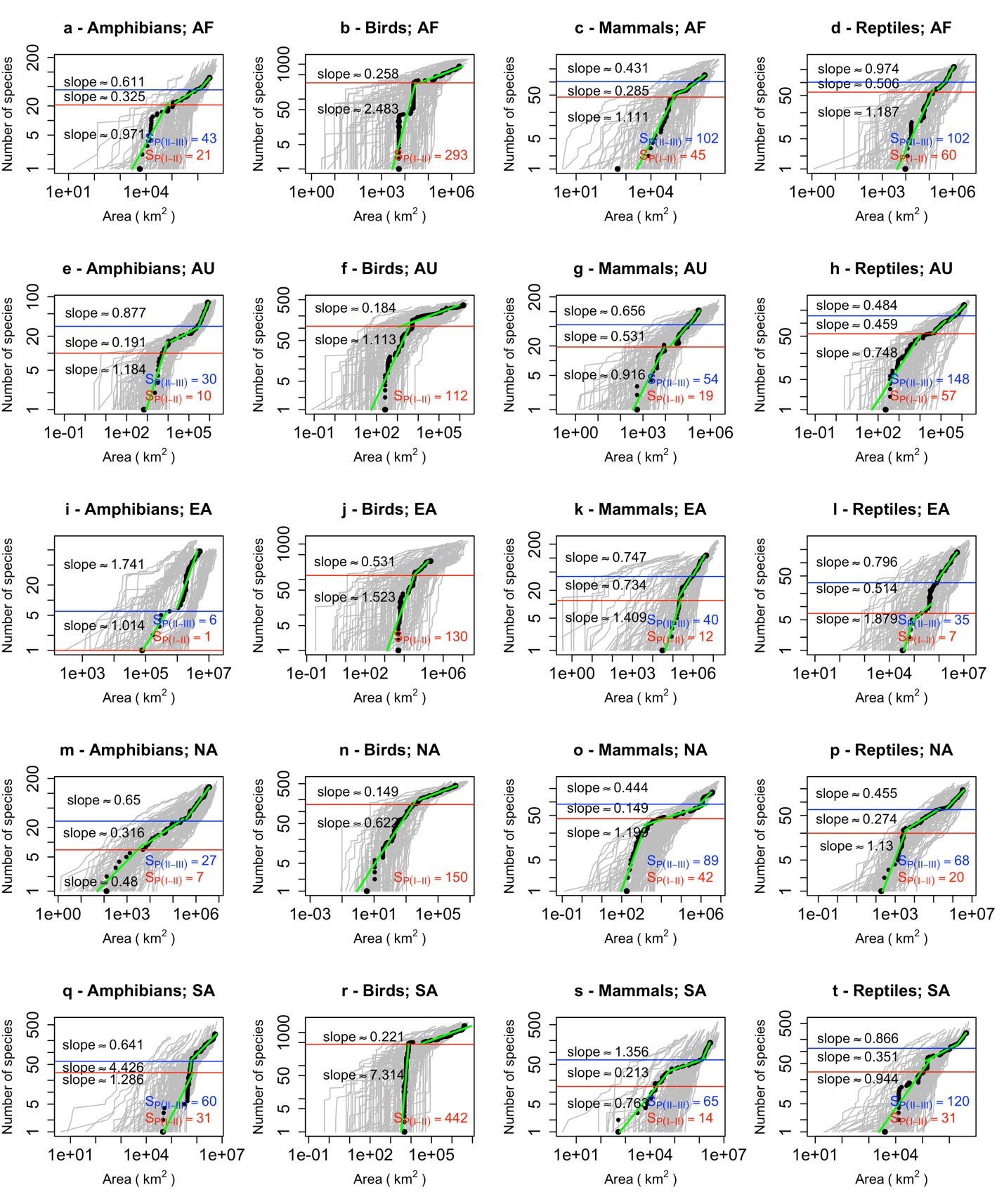
Fig. S13 |** **Empirical GBIF SARs alongside corresponding *S_P_* predictions*.*** The grey curves in the background represent SARs obtained from 200 randomly located focal points, while the black dots indicate a mean SAR (see Methods for details). The horizontal red lines represent the number of species predicted at the transition between Phases I and II, *S_P_*_(_*_I-II_*_)_, and the horizontal blue lines represent the predicted number between Phases II and III, *S_P_*_(_*_II-III_*_)_.

**
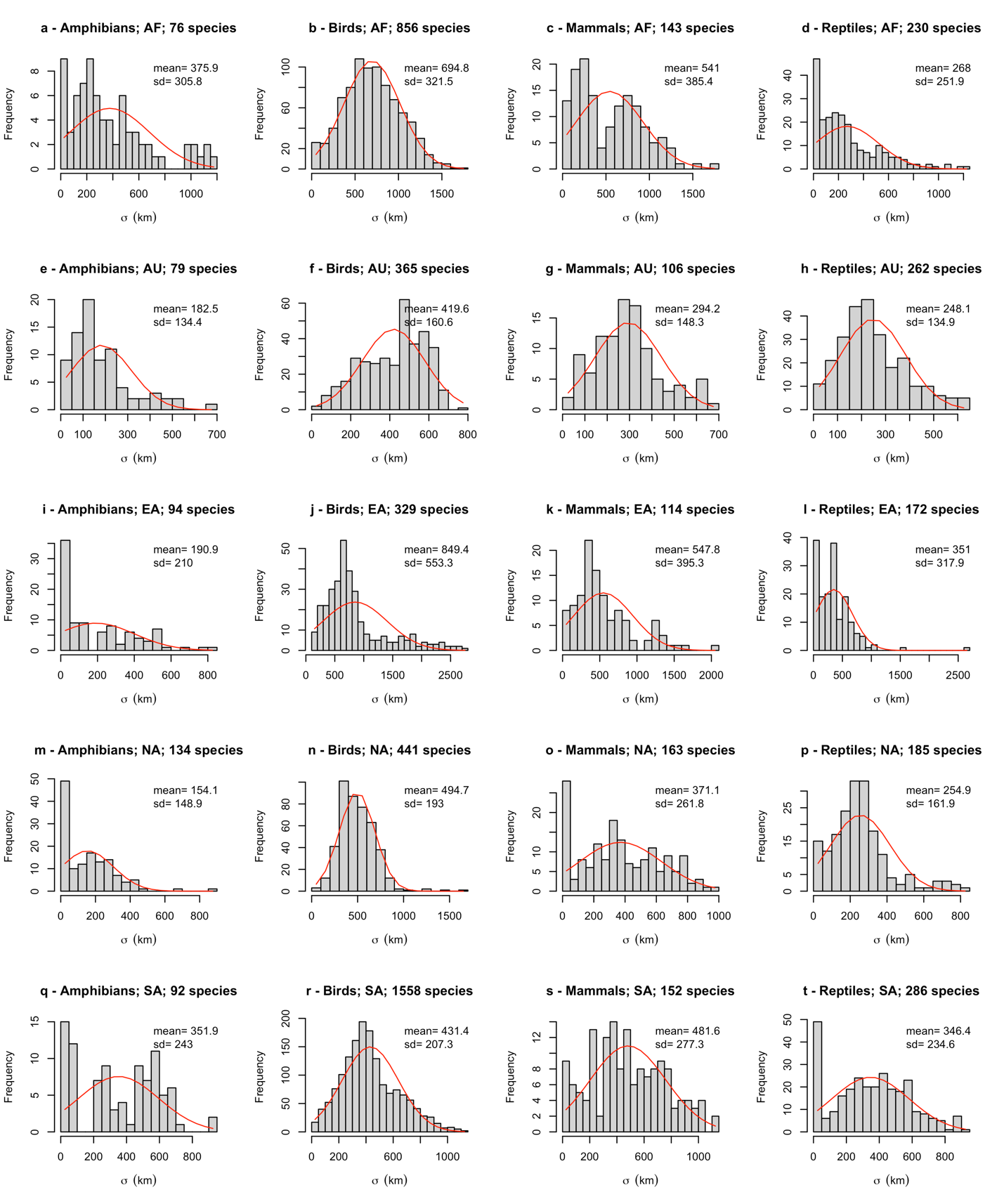
**

**Fig. S14 | Histograms of the width of the distributions of distances of the individuals to the centre of the range.** The red curves show fitted normal distributions.

Table S2 | The values of *S_P_*_(I-II)_ and *S_P_*_(II-III)_ corresponding to SARs in black dots in Fig. S13. There is no Phase 3 for birds, hence, there are no *S_P_*_(II-III)_ values. The numbers in brackets correspond to the fraction to the total number of species.

|  | | Amphibians | Birds | Mammals | Reptiles |
| --- | --- | --- | --- | --- | --- |
| Africa | *S_P_*_(I-II)_ | 21 (0.28) | 293 (0.34) | 45 (0.31) | 60 (0.26) |
|  | *S_P_*_(II-III)_ | 43 (0.56) | - | 102 (0.71) | 102 (0. 44) |
| Australia | *S_P_*_(I-II)_ | 10 (0.13) | 112 (0.31) | 19 (0.18) | 57 (0.22) |
|  | *S_P_*_(II-III)_ | 30 (0.38) | - | 54 (0. 51) | 148 (0.56) |
| Eurasia | *S_P_*_(I-II)_ | 1 (0.01) | 130 (0.40) | 12 (0.10) | 7 (0.04) |
|  | *S_P_*_(II-III)_ | 6 (0.06) | - | 40 (0.35) | 35 (0.20) |
| North America | *S_P_*_(I-II)_ | 7 (0.05) | 150 (0.34) | 42 (0.26) | 20 (0.11) |
|  | *S_P_*_(II-III)_ | 21 (0.20) | - | 89 (0.55) | 68 (0.37) |
| South America | *S_P_*_(I-II)_ | 31 (0.11) | 442 (0.28) | 14 (0.08) | 31 (0.11) |
|  | *S_P_*_(II-III)_ | 60 (0.21) | - | 65 (0.37) | 120 (0.42) |

**Table S3 |** **The slopes of the several phases in Fig. S13 (the green lines).** We show in red the case where the slope estimated for Phase II is larger than the slopes estimates to the other two phases.

|  | | Amphibians | Birds | Mammals | Reptiles |
| --- | --- | --- | --- | --- | --- |
| Africa | Phase I | 0.971 | 2.483 | 1.111 | 1.187 |
|  | Phase II | 0.325 | 0.256 | 0.2854 | 0.506 |
|  | Phase III | 0.611 | - | 0.431 | 0.974 |
| Australia | Phase I | 1.184 | 1.113 | 0.916 | 0.748 |
|  | Phase II | 0.191 | 0.184 | 0.531 | 0.459 |
|  | Phase III | 0.877 | - | 0.656 | 0.484 |
| Eurasia | Phase I | - | 1.523 | 1.409 | 1.879 |
|  | Phase II | 1.014 | 0.531 | 0.734 | 0.514 |
|  | Phase III | 1.741 | - | 0.747 | 0.796 |
| North America | Phase I | 0.480 | 0.622 | 1.199 | 1.130 |
|  | Phase II | 0.316 | 0.149 | 0.149 | 0.274 |
|  | Phase III | 0.650 | - | 0.444 | 0.455 |
| South America | Phase I | 1.286 | 7.314 | 0.763 | 0.955 |
|  | Phase II | 4.426 | 0.221 | 0.213 | 0.351 |
|  | Phase III | 0.641 | - | 1.356 | 0.866 |

**References**

1. Arnold, B. C., Balakrishnan, N. & Nagaraja, H. N. *A First Course in Order Statistics*. vol. 54 (Siam, 2008).

2. Galambos, J. *The Asymptotic Theory of Extreme Order Statistics, Second Edition*. (Robert E. Krieger Publishing Company, 1987).

3. Coles, S. *An Introduction to Statistical Modeling of Extreme Values*. (Springer, 2001).

4. Castillo, E., Hadi, A. S., Balakrishnan, N. & Sarabia, J. M. *Extreme Value and Related Models with Applications in Engineering and Science*. (Wiley, 2005).

5. Fréchet, M. Sur la loi de probabilité de l’écart maximum. *Ann Soc Pol. Math* **6**, (1927).

6. Fisher, R. A. & Tippett, L. H. C. Limiting forms of the frequency distribution of the largest or smallest member of a sample. *Proc. Camb. Philos. Soc.* **24**, 180–190 (1928).

7. Gumbel, E. J. Les valeurs extrêmes des distributions statistiques. *Ann. Inst. Henri Poincaré* **5**, 115–158 (1935).

8. Kobayashi, H., Mark, B. L. & Turin, W. *Probability, Random Processes, and Statistical Analysis: Applications to Communications, Signal Processing, Queueing Theory and Mathematical Finance*. (CUP, 2012).

9. Allen, A. P. & White, E. P. Effects of range size on species-area relationships. *Evol. Ecol. Res.* **5**, 493–499 (2003).

10. Bocus, M. Z., Dettmann, C. P. & Coon, J. P. An approximation of the first order Marcum Q-function with application to network connectivity analysis. *IEEE Commun. Lett.* **17**, 499–502 (2013).
